# A simplified method for monitoring cytokines in wound fluid

**DOI:** 10.1111/wrr.13053

**Published:** 2022-10-26

**Authors:** Ewa Anna Burian, Christian Enevold, Tonny Karlsmark, Magnus S. Ågren

**Affiliations:** ^1^ Department of Dermato‐Venereology and Wound Healing Center, Bispebjerg Hospital University of Copenhagen Copenhagen Denmark; ^2^ Institute for Inflammation Research, Center for Rheumatology and Spine Diseases, Rigshospitalet University Hospital Copenhagen Denmark; ^3^ Department of Clinical Medicine, Faculty of Health and Medical Sciences University of Copenhagen Copenhagen Denmark; ^4^ Digestive Disease Center, Bispebjerg Hospital University of Copenhagen Copenhagen Denmark

**Keywords:** biomarkers, inflammation, leg ulcer, swab, wound fluid collection

## Abstract

Cytokines in wound fluid are used as surrogates for wound healing in clinical research. The current methods used to collect and process wound fluid are noninvasive but not optimal. The aim of this prospective study was to evaluate a method (NovaSwab) by which wound fluid is collected by a surface swab and eluted in a physiological buffer for subsequent cytokine analysis. Wound fluid from 12 patients with leg ulcers was assessed by NovaSwab at the start (Day 0) and at the end of a 23‐h collection period of wound fluid retained by foam oblates beneath an occlusive film dressing (Day 1). GM‐CSF, IL‐1α, IL‐1β, IL‐6, IL‐8, PDGF‐AA, TNF‐α and VEGF levels were measured by multiplex and electrochemiluminescence assays. IL‐1α (2.4×), IL‐1β (2.0×) and IL‐8 (1.8×) levels increased from Day 0 to Day 1 as detected by NovaSwab, indicating local production of these polypeptides in the wounds. On Day 1, the NovaSwab method yielded higher levels of IL‐1α (4.0×), IL‐1β (2.7×) and IL‐6 (2.7×), and 35% lower levels of VEGF than those in wound fluid accumulated for 23 h in foam oblates (on average, 5 ml of wound fluid). In vitro experiments showed that the investigated cytokines in cell‐free wound fluid were recovered in a quantitative manner by the NovaSwab method*.* We conclude that the method presented here is a promising research tool to study the kinetics of soluble cytokines over the course of wound healing. More studies are needed to determine the interobserver variation and reproducibility of the NovaSwab method.

**Abbreviations**
GM‐CSF: granulocyte‐macrophage colony‐stimulating factor
IL: interleukin
PDGF: platelet‐derived growth factor
SD: standard deviation
TNF: tumour necrosis factor
VEGF: vascular endothelial growth factor
WF: wound fluid

## 1 INTRODUCTION

Wound fluid (WF) is commonly used for studies of the cytokines and growth factors involved in wound healing. WF mirrors the local microenvironment well and is easily accessible.^1^, ^2^

There has been a call for researchers to standardise the methods for WF collection to accurately measure cytokines, but there is a paucity of simple, rapid and reliable methods for WF collection.^2^, ^3^, ^4^ Aspiration of WF accumulating under occlusive film dressings is a common technique in leg ulcers.^4^, ^5^, ^6^, ^7^, ^8^, ^9^, ^10^, ^11^ Drawbacks with this technique are leakage of WF and an insufficient amount of procured WF, especially from dry and small wounds.^11^ To increase the amount of WF generated, Trengove et al. had their leg ulcer patients drink 1 L water in the morning and WF was collected after 1 h of leg dependency.^12^ WF has also been collected and extracted from polyurethane foam and other dressing types^5^, ^13^, ^14^, ^15^, ^16^, ^17^, ^18^ and by topical negative wound therapy pressure devices.^19^ Especially for longitudinal studies with repetitive measurements, these time‐consuming methods have been problematic. In one study, less than half of the patients produced sufficient amounts of WF for sequential measurements.^11^

With the introduction of more sensitive analytic techniques, the demand for WF volumes has gradually decreasing, stimulating the development of simpler sampling methods.^20^, ^21^, ^22^, ^23^ For example, Schmohl et al.^21^ introduced WF collection with nylon surface swabs from diabetic foot ulcers. WF (~40 μl) was retrieved by centrifuging the swab in an insert in a microcentrifuge tube and freezing it.^21^ The technique yielded satisfying results compared with WF collected by aspiration although IL‐1α, IL‐1β and TNF‐α levels tended to be elevated. However, freezing of naïve WF may disrupt cells and release their cytokine content.^24^ Furthermore, to overcome the disadvantage of the small WF volumes collected, we have developed the NovaSwab method. In brief, WF is obtained from the wound by a surface swab and eluted directly by a physiological buffer in a microcentrifuge tube that is vortexed. Cells and cell debris are removed by centrifugation and the supernatant, representing the extracellular compartment, is analysed.

Zillmer et al.^5^ developed and validated a WF collection method using a collection device (hydrophobic polyurethane foam) combined with an occlusive film dressing to avoid WF leakage. The foam material did not influence the levels of the investigated cytokines.^5^ Interestingly, the authors demonstrated a time‐dependent increase in several cytokines over 24 h using this method in venous leg ulcers.^5^

Our objective was to compare the NovaSwab method to the Zillmer method^5^ primarily by reproducing the dynamic cytokine changes in patients with leg ulcers. The effect of wound irrigation with saline on cytokine levels was investigated with the NovaSwab method. The NovaSwab method was further validated by studying the recovery of cytokines in WFs in vitro. All samples were analysed for eight cytokines selected on the basis of the results of a recent systematic review of cytokines in venous leg ulcer healing.^25^

## 2 MATERIALS AND METHODS

### 2.1 Study design

This prospective, exploratory study involved two sessions (Day 0 and Day 1) for the patients. The study was approved by the Ethics Committee of the Capital Region (H‐19081985) and conducted at the Copenhagen Wound Healing Center, Department of Dermatology, University of Copenhagen, Copenhagen, Denmark in accordance with the Declaration of Helsinki.

### 2.2 Participants and ulcer area determination

Hospitalised patients (*n* = 8) and outpatients (*n* = 4) with chronic leg ulcers who fulfilled all of the selection criteria were consecutively recruited from our department, a tertiary health care centre. The inclusion criteria were patients aged ≥18 years old, with at least one nonhealing leg ulcer of a duration of ≥2 months, localised between the ankle and the knee (including the perimalleolar area); being informed about the study; being able to comply with the protocol and having given oral and written informed consent. The exclusion criteria were allergies to any of the materials used in the study, the index ulcer judged as having insufficient production of WF, any major changes in antibiotics or immunosuppressive treatment over the previous 7 days, or planned changes during the study. These changes included any initiation, discontinuation or major dosage changes in this treatment. Pregnant women and patients judged unsuitable to participate, for example, patients receiving palliative care, were excluded.

The ulcer area was determined by ImageJ software (ImageJ 1.49, NIH, Bethesda, MD) from digitised tracings of the epidermal edge on an acetate film.

### 2.3 The NovaSwab method (Figure 1)

**FIGURE 1:**
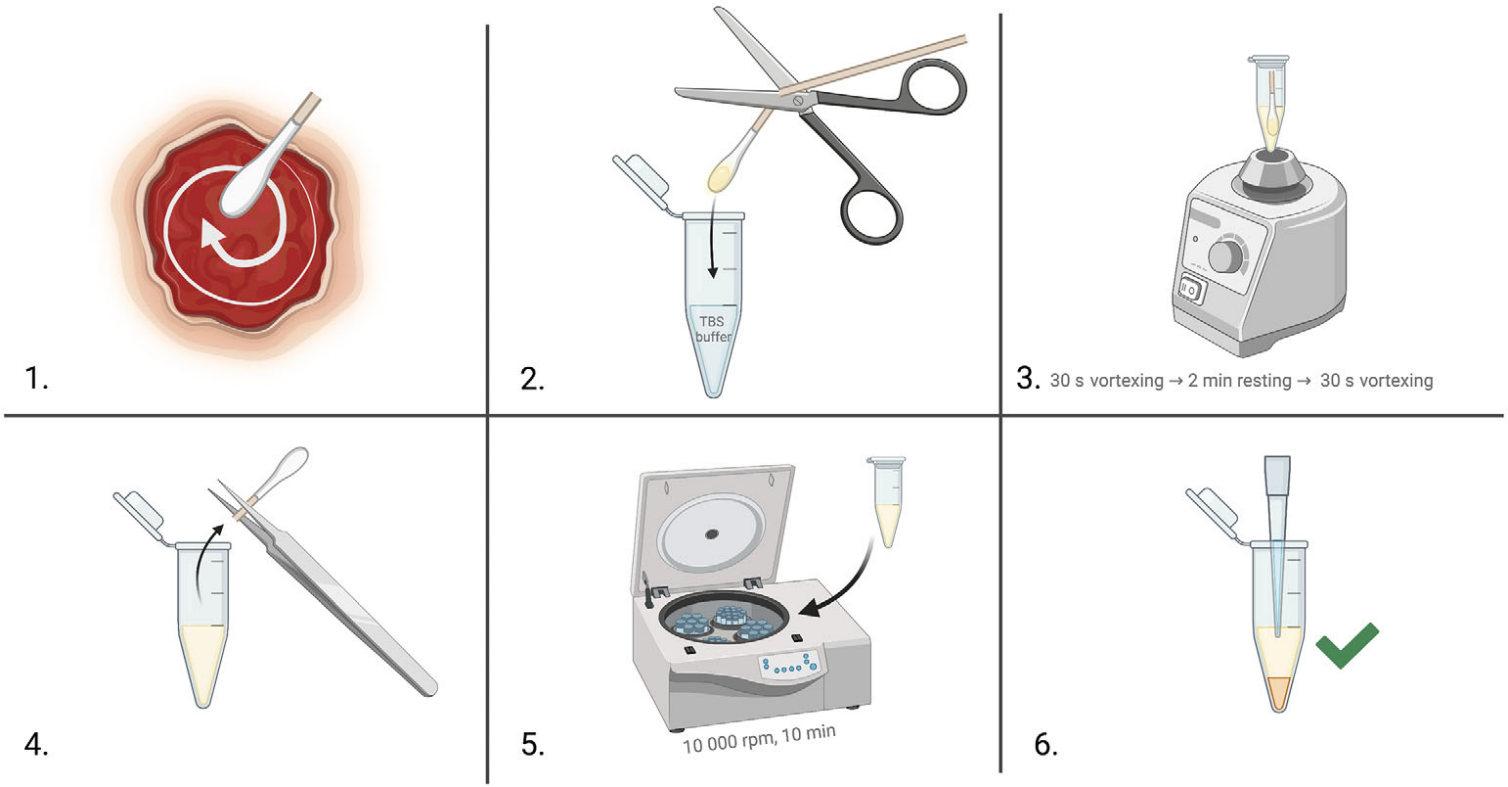
The six‐step NovaSwab method for the measurement of cytokines in WF collected with a surface swab

WF was collected by a sterile swab that was gently pressed to the wound bed in a spiral manner starting at the edge and ending at the wound centre according to the Essen Rotary technique.^26^ The swab tip was amputated to 2 cm and placed in a 1.5‐ml microcentrifuge polypropylene tube (616,261, Greiner Bio‐One GmbH, Frickenhausen, Germany or BioSphere® SafeSeal, 72.706.200, Sarstedt, Nümbrecht, Germany) containing 300 μl of Tris‐buffered saline (TBS; 10 mmol/L Tris and 0.9% NaCl, pH 7.4), which was vortexed at 2700 rpm (Vortex‐Genie 2, Scientific Industries, Bohemia, NY) for 30 s and again after 2 min. The tip of the swab was pressed gently against the upper, liquid‐free part of the tube wall and discarded. The tube was centrifuged at 8000*g* (10,000 rpm) for 10 min at 4°C (Centrifuge 5402, Eppendorf, Engelsdorf, Germany), and the supernatant was stored at −80°C.

### 2.4 WF sampling schedule and procedures including WF procured by the Zillmer method^5^

**FIGURE 2:**
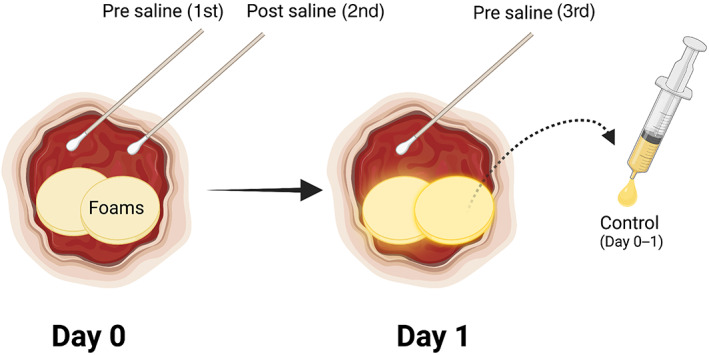
Overview of WF samples collected from each patient. On Day 0, WF was obtained with surface swabs before (1st swab) and after saline irrigation (2nd swab). Foam oblates were then applied to the wound bed and the wound was covered with an occlusive film dressing according to Zillmer et al.^5^ On Day 1, WF was squeezed out from the foam oblates and centrifuged to yield a cell‐free supernatant (controls in the in vitro tests). Finally, WF was collected with a 3rd swab.

An overview of the WF samples collected from each patient is shown in Figure 2.

On Day 0, WF was obtained with a surface swab before (1st swab) irrigation with sterile 0.9% NaCl (saline) from a 10‐ml prefilled syringe (BD PosiFlush™ SP syringe, Becton Dickinson, Franklin Lakes, NJ). Excessive saline was dabbed dry with nonwoven compress before swabbing with a 2nd swab.

WF was then collected according to Zillmer et al.^5^ Eight‐millimetre thick hydrophobic polyurethane foam oblates (SteriSets Medical Products, Penafiel, Portugal) were applied to the wound bed, a skin barrier film was applied to the surrounding skin (Silesse™, ConvaTec, Deeside, UK) and the wound with the foam oblates was covered with an occlusive film dressing (Tegaderm™ Film, 3M™, St. Paul, MN). The foam oblates were removed after 20–25 h and placed in 10‐ to 30‐ml syringes with the plungers removed, and WF was squeezed out and centrifuged (8000*g* for 10 min at 4°C). The WF volume was determined by pipetting.

Finally, WF was collected from the wound bed with a 3rd surface swab on Day 1.

### 2.5 Uptake of WF by surface swabs

Two brands of surface swabs were used: eSwab (BD, Copan Italia, Brescia, Italy) with a tip flocked with soft nylon and ∑‐Transwab® (MWE, Corsham, UK) with a polyurethane open‐cell foam tip. The uptake (μl) of WF from the wounds on Day 1 by eSwab (*n* = 6) and ∑‐Transwab (*n* = 6) was estimated using the formula (assuming 100% elution of the proteins from the swabs): 300/(([protein_WF_]/[protein_Sample_]) − 1).

### 2.6 In vitro studies

The recovery of cytokines in WF by the NovaSwab and Schmohl methods was studied (Figure S1A). Cell‐free WF collected by foam oblates^5^ (50 μl) from all patients was added to the tip of swabs (∑‐Transwab [*n* = 11] and eSwab [*n* = 1]) and absorbed for 30 s; one swab was processed according to the NovaSwab method (steps 2–4, Figure 1) and the other swab according to a modified method by Schmohl et al.,^21^ that is, the swab was amputated to a length of 1 cm and the swab tip centrifuged (8000*g* for 3 min at 4°C) in a 1.5‐ml microcentrifuge tube with a centrifugal filter unit (Ultrafree®‐MC, Millipore) without the filter membrane. The amputated swab and the filter unit were subsequently discarded, and the WF was transferred to a 1.5‐ml tube (616,261, Greiner Bio‐One or 72.706.200, Sarstedt).

The recovery of proteins by the NovaSwab method was estimated. Cell‐free WF (50 μl) from three patients was added to the tip of ∑‐Transwabs and absorbed for 30 s, and the tip was cut off and placed in a 1.5‐ml tube (616,261, Greiner Bio‐One or 72.706.200, Sarstedt) containing 300 μl of TBS and processed according to the NovaSwab (steps 3–4, Figure 1) method (1st extraction). The tip of the swab was transferred to a new 1.5‐ml tube containing 300 μl of TBS, and the extraction was repeated (2nd extraction). The protein concentrations of the two extracts were measured (mg/ml). The recovery of total proteins (%) was calculated using the formula: [protein_1st extraction_]/([protein_1st extraction_] + [protein_2nd extraction_]) × 100.

### 2.7 Determination of cytokines and total proteins

The concentrations of IL‐1α, IL‐1β, IL‐6, IL‐8, PDGF‐AA, TNF‐α and VEGF‐A were determined using a bead‐based multiplexed immunoassay system (Luminex® Discovery Assay, Human Premixed Multianalyte Kit, R&D Systems, Minneapolis, MN) on a Bio‐Plex 200 system (Bio‐Rad Laboratories, Hercules, CA) as described by the manufacturers. Reported sensitivities for each analyte are reported in Table S1. Samples were serially diluted in the assay calibrator diluent (RD6‐52) starting at a twofold dilution. Spike‐in of the cytokines into two randomly selected WF samples showed recoveries from 80% to 133%. GM‐CSF was measured by an electrochemiluminescence assay kit (S‐PLEX, Meso Scale Discovery, Rockville, MD) and luminescence was read on an MESO® QuickPlex SQ 120. The reported sensitivity of GM‐CSF is shown in Table S2. The total protein content of the samples was measured using a modified Lowry assay (DC Protein Assay Kit 2, Bio‐Rad Laboratories). Samples and bovine serum albumin standard (Bio‐Rad Laboratories) were diluted in distilled water and 5 μl of diluted samples, standard dilutions (0.196–1.49 mg/ml) and blank (0 mg/ml) were added to a 96‐well plate. The assay was run according to the manufacturer's recommendations, and optical densities were read at 650 nm on a microplate reader (Multiskan FC, Thermo Scientific, Singapore).

### 2.8 Statistical analysis

Because it was not possible to estimate the sample size, we decided to include 12 patients. Since the data were not normally distributed for cytokines, logarithmic transformations were performed (log_10_). Two‐tailed paired and unpaired *t*‐tests were used in comparative analyses. Bland–Altman plots were created to visualise the agreement in cytokine levels in WF determined by the NovaSwab and Zillmer^5^ methods. Spearman's correlation analysis was applied. Data are presented as the mean ± SD or differences with 95% confidence intervals (CI) unless otherwise stated. Statistical significance was set to *p* < .05. GraphPad Software (Version 9.3.1, San Diego, CA) was used for statistical analyses.

## 3 RESULTS

### 3.1 Participants, wound characteristics, treatments, WF production and reference cytokine levels

Twelve patients (73 ± 10 years old, range: 57–89 years), six men and six women, were included between May 2020 and October 2021. Six patients had venous leg ulcers, two had pyoderma gangrenosum, one had an arterial ulcer, one patient had calciphylaxis, one was described as idiopathic/inflammatory and one patient had Martorell's ulcer. Ten patients had palpable foot pulses, and ischemia was suspected in one patient.

Two patients had multiple adjacent ulcers. The sum of these ulcer areas was used for the calculation of the mean ulcer area of the 12 patients (22 ± 21 cm^2^, range: 5.7–76 cm^2^). The mean ulcer duration was 15 ± 12 months (range: 2.5–48 months). No ulcers showed signs of clinical infection.

Before inclusion, an active wound dressing (Biatain® Ibu, Coloplast, Humlebæk, Denmark [*n* = 2], silver [*n* = 3] or combination of Biatain® Ibu and silver [*n* = 2]) was used in seven patients, and a neutral standard dressing was used in five patients. All patients received compression therapy using multilayer or elastic bandages at inclusion and during WF collection with the foam oblates. Two adverse events were registered: skin maceration was noted in one patient, and another patient experienced an allergic reaction to the film dressing.

WF was collected with foam oblates (4.9 ± 4.3 ml, range: 0.3–12 ml) over the course of 23 ± 1.5 h; this process translated into an estimated WF production rate of 0.25 ± 0.14 ml/cm^2^/day. The WF volume correlated strongly with the wound surface area (*r*_*S*_ = 0.87, *p* = .0005).

Cytokine levels in pg/ml in WF collected by the foam oblates are presented in Table 1.

**TABLE 1:** Cytokine concentrations (pg/ml) in WF collected by foam oblates for 23 h

Cytokine	Mean ± SD	Median (IQR)	Range	*n*
GM‐CSF	360 ± 950	21 (4.9–65)	0.51–3200	11
IL‐1α	4200 ± 3100	3800 (1800–6900)	340–11,000	12
IL‐1β	52,000 ± 44,000	31,000 (23,000–94,000)	16,000–150,000	11
IL‐6	92,000 ± 260,000	8200 (2400–28,000)	420–920,000	12
IL‐8	470,000 ± 390,000	350,000 (190,000–700,000)	79,000–1,300,000	10
PDGF‐AA	520 ± 1200	110 (71–290)	68–3800	10
TNF‐α	2300 ± 2200	2000 (500–3000)	220–8100	12
VEGF	9700 ± 3100	9800 (6900–12,000)	5300–17,000	12

Abbreviations: IQR, interquartile range; SD, standard deviation. *n*, number of patients = number of WF samples.

### 3.2 In vivo assessments of the NovaSwab method

Due to backorders during the COVID‐19 pandemic, ∑‐Transwab replaced eSwab for WF collection from the last six patients. WF uptake from the wounds was similar (*p* = .87) for eSwab (53 ± 24 μl) and ∑‐Transwab (50 ± 31 μl). The results of the cytokine analyses of WFs obtained by eSwab and ∑‐Transwab were aggregated for the statistical analyses.

Saline irrigation of the wound increased the levels of IL‐1α by 1.8 times (95% CI: 1.1–2.8, *p* = .016) and IL‐8 by 1.7 times (95% CI: 1.2–2.5, *p* = .0091) compared with before irrigation (Table 2 and Figure S2). In contrast, IL‐6 levels decreased by 23% (95% CI: 3.5%–39%, *p* = .027) after saline irrigation. The total protein concentration of WF processed by NovaSwab was 5.4 ± 3.6 mg/ml before (1st swab) and 3.2 ± 1.6 mg/ml after (2nd swab) saline irrigation (*p* = .0061).

**TABLE 2:** Cytokine levels normalised to the total protein in WF determined by the NovaSwab method (log_10_) sampled at 3 occasions Day 0 and Day 1

Cytokine	Day 0		Day 1
	Pre saline (1st swab)^a^	Post saline (2nd swab)^a^	Pre saline (3rd swab)^a^
GM‐CSF (fg/mg)	3.11 ± 0.71 (*n* = 6)	3.23 ± 0.59 (*n* = 6)	3.22 ± 0.80 (*n* = 9)
IL‐1α (pg/mg)	2.06 ± 0.31 (*n* = 12)	2.32 ± 0.36 (*n* = 12)	2.44 ± 0.44 (*n* = 12)
IL‐1β (pg/mg)	3.11 ± 0.42 (*n* = 11)	3.19 ± 0.42 (*n* = 11)	3.41 ± 0.52 (*n* = 11)
IL‐6 (pg/mg)	2.54 ± 0.69 (*n* = 12)	2.43 ± 0.67 (*n* = 12)	2.78 ± 0.74 (*n* = 12)
IL‐8 (pg/mg)	3.83 ± 0.27 (*n* = 10)	4.07 ± 0.32 (*n* = 10)	4.01 ± 0.37 (*n* = 10)
PDGF‐AA (pg/mg)	0.78 ± 0.66 (*n* = 6)	1.22 ± 0.35 (*n* = 5)	0.59 ± 0.45 (*n* = 10)
TNF‐α (pg/mg)	1.20 ± 0.46 (*n* = 10)	1.29 ± 0.43 (*n* = 11)	1.44 ± 0.58 (*n* = 12)
VEGF (pg/mg)	2.21 ± 0.24 (*n* = 12)	2.13 ± 0.24 (*n* = 12)	2.15 ± 0.24 (*n* = 12)

*Note*: Mean ± SD. *n*, number of patients = number of WF samples.

^a^ See Figure 2 for explanations.

Cytokine levels determined at baseline Day 0 (1st swab) and 23 h later on Day 1 (3rd swab) by the NovaSwab method were compared; IL‐1α was 2.4 times (95% CI: 1.4–4.0, *p* = .0041) higher, IL‐1β was 2.0 times (95% CI: 1.0–3.9, *p* = .048) higher, and IL‐8 was 1.8 times (95% CI: 1.0–3.3, *p* = .044) higher on Day 1 versus Day 0 (Table 2).

**FIGURE 3:**
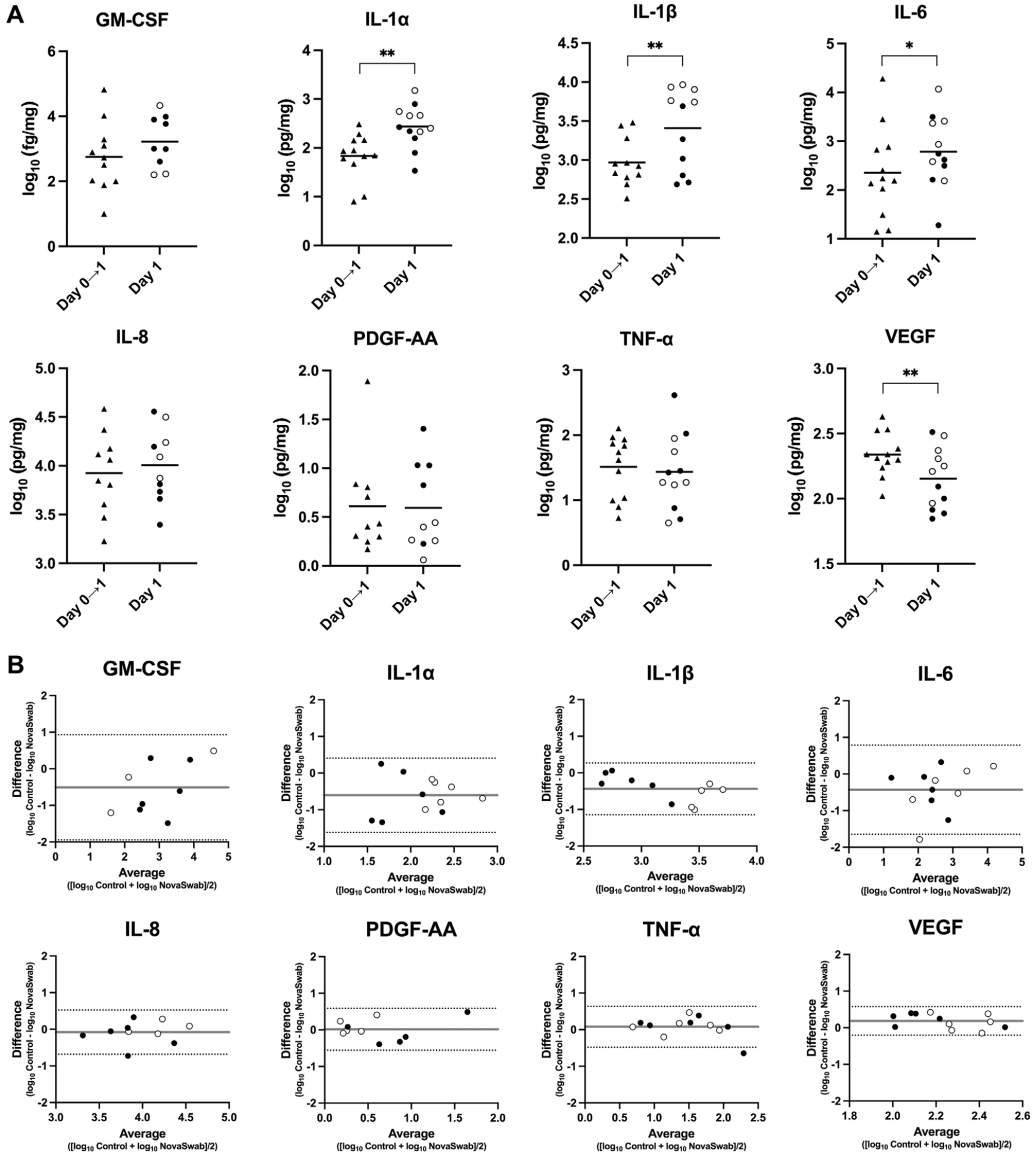
Cytokine levels normalised to the total protein measured in WF accumulating from Day 0 to Day 1 for 23 h in foam oblates (Day 0 → 1)^5^ and by the NovaSwab method (Day 1). (A) Individual and mean cytokine values from the 12 patients. *p < .05; **p < .01. Triangles, WF collected by foam oblates; open circles, WF samples obtained by eSwab (first 6 patients); filled circles, ∑‐Transwab (last 6 patients). (B) Bland–Altman plots of agreement between cytokine levels in WF determined with the NovaSwab method on Day 1 and WF accumulating in foam oblates from Day 0 to Day 1 (control). The mean bias is indicated by the horizontal grey lines, and the 95% limits of agreement are indicated by the dotted horizontal lines.

On Day 1, the NovaSwab method yielded 4.0 times (95% CI: 1.9–8.6, *p* = .0019) higher levels of IL‐1α, 2.7 times (95% CI: 1.6–4.8, *p* = .0024) higher levels of IL‐1β and 2.7 times (95% CI: 1.1–6.7, *p* = .036) higher levels of IL‐6 than those in WF accumulating for 23 h in foam oblates. VEGF WF levels were 35% (95% CI: 13%–51%, *p* = .0079) lower with NovaSwab than with foam oblates. Bland–Altman plots showed acceptable agreement between the NovaSwab compared with WF collected by foam oblates. The 95% limits of agreement were broadest for GM‐CSF and narrowest for VEGF (Figure 3B). WFs collected by eSwab and ∑‐Transwab respectively are indicated in Figure 3. The total protein concentration of WF on Day 1 processed by NovaSwab was 5.9 ± 2.2 mg/ml (3rd swab).

### 3.3 In vitro studies

WF cytokine levels were studied after being subjected to the NovaSwab and Schmohl methods (Figure S1A). In comparison with the controls (no contact with swabs), NovaSwab did not alter the concentrations of the eight investigated cytokines in the 12 different WF samples (Table 3 and Figure S1B). Compared with Schmohl, the NovaSwab method increased GM‐CSF by 1.6 times (95% CI: 1.1–2.4, *p* = .018), IL‐1α by 1.1 times (95% CI: 1.0–1.3, *p* = .032) and TNF‐α by 1.1 times (95% CI: 1.0–1.3, *p* = .013). IL‐6 levels were 23% (95% CI: 12%–33%, *p* = .0015) lower with NovaSwab than with the Schmohl method. The total protein concentration of the NovaSwab samples was 6.3 ± 1.6 mg/ml (total protein in WF was recovered to 77% ± 1.5% for three WF samples), the WF processed with the Schmohl method was 50 ± 12 mg/ml, and it was 43 ± 10 mg/ml for the controls.

**TABLE 3:** Recovery of cytokines normalised to the total protein by NovaSwab method (log_10_) in vitro

	Control	NovaSwab	*n*	Mean difference	95% CI	*p* ^a^
GM‐CSF (fg/mg)	2.75 ± 1.06	2.93 ± 0.90	11	−0.17	−0.37 to 0.027	.082
IL‐1α (pg/mg)	1.83 ± 0.47	1.87 ± 0.51	12	−0.035	−0.12 to 0.047	.37
IL‐1β (pg/mg)	2.97 ± 0.31	2.98 ± 0.42	11	−0.012	−0.15 to 0.12	.84
IL‐6 (pg/mg)	2.36 ± 0.92	2.27 ± 0.91	12	0.082	−0.012 to 0.18	.080
IL‐8 (pg/mg)	3.93 ± 0.42	3.92 ± 0.51	10	0.0087	−0.19 to 0.21	.93
PDGF‐AA (pg/mg)	0.64 ± 0.53	0.66 ± 0.40	9	−0.012	−0.13 to 0.11	.83
TNF‐α (pg/mg)	1.52 ± 0.48	1.49 ± 0.49	12	0.024	−0.067 to 0.12	.57
VEGF (pg/mg)	2.34 ± 0.17	2.33 ± 0.22	12	0.0095	−0.062 to 0.081	.78

*Note*: Mean ± SD. *n*, number of patients = number of WF samples.

^a^ Paired *t*‐test.

## 4 DISCUSSION

There is a need for convenient and reliable methods to collect WF for cytokine analysis in longitudinal clinical studies of wound healing. Our results support that the NovaSwab method presented here recovers cytokines in collected WFs in a representative manner. Moreover, we confirmed the findings by Zillmer et al.,^5^ demonstrating increased WF levels of IL‐1α, IL‐1β and IL‐8 over time in leg ulcers. Considered together, these findings indicate that the NovaSwab method could be used to monitor cytokine changes over time.

The cells responsible for the secretion of cytokines have been only sparsely described in the literature on leg ulcers.^27^, ^28^, ^29^, ^30^ In acute wounds, keratinocytes are prominent cytokine producers at the wound edge^31^, ^32^ and of macrophages and fibroblasts in the wound bed.^33^, ^34^ The senescent fibroblast phenotype commonly found in nonhealing leg ulcers contributes to the proinflammatory environment.^35^, ^36^, ^37^ Although TNF‐α immunostaining is strong in chronic venous leg ulcers compared with healing venous leg ulcers and normal skin,^28^, ^30^ TNF‐α levels did not increase in WF over 23 h in our study. GM‐CSF, IL‐6, PDGF‐AA and VEGF levels also did not change as a function of time. We cannot exclude that these findings could be ascribed to the diverse aetiologies of the non‐healing leg ulcers and/or different wound dressings used prior inclusion. However, the TNF‐α and VEGF levels, as well as those of IL‐1α, IL‐1β and IL‐8, found here were similar to those reported for other cohorts of venous leg ulcer patients using the same collection method.^5^, ^38^, ^39^

We had to change the swab supplier in the middle of the study. The two swab types, one with a soft nylon tip and the other with a polyurethane foam tip, were comparable in WF uptake and did not show apparent systematic differences in cytokine levels. Importantly, the same swab type was used for each patient, enabling paired comparisons (Table 2).

We chose the Essen Rotary technique for WF collection to account for local variations in cytokine abundances in wounds (step 1, Figure 1).^23^, ^26^, ^27^, ^31^, ^40^, ^41^ The alternative Levine technique might be biased because only the centre of the wound is sampled.^42^

Another possible source of variation is the use of irrigation solutions.^43^, ^44^ Unexpectedly, we found significant increases in IL‐1α and IL‐8, while IL‐6 decreased after saline irrigation. These findings might reflect differences in the affinity of cytokines for cell receptors and extracellular matrix components. Whatever the mechanisms, we recommend that WF be collected before irrigating the wound with saline or other solutions because this WF is genuine and has not been manipulated.

The cytokine measurements with the NovaSwab method were compared with those measured in WF that had accumulated in foam oblates covered with an adhesive polyurethane film dressing.^5^ This method secured sufficient WF for the in vitro tests. Elicitation of adverse skin reactions by the film dressing is problematic for repetitive sampling of WF.^5^ The levels of IL‐1α, IL‐1β and IL‐6 were higher, but those of VEGF were lower with NovaSwab than with WF accumulating for 23 h in foam oblates. Foam oblates have been validated in venous leg ulcers for IL‐1α, IL‐β and IL‐8 but not for VEGF^5^; hence, we cannot exclude the possibility that foam oblates per se influence VEGF levels in leg ulcers. Admittedly, the Zillmer method^5^ might not provide a realistic snapshot of cytokine concentrations in wounds. The cytokine levels in WF reflect the net results of several concurrent processes in which secretion and degradation are important.^29^ VEGF secretion might have declined over the 23‐h collection period.

Differences in susceptibilities to proteolysis of accumulated cytokines provide an additional or alternative explanation. The half‐lives of cytokines are generally short.^45^ The half‐life of parenteral rhIL‐1β was <20 min in the serum of rats.^46^ Boink et al.^47^ found no significant degradation of rhIL‐6 (500 μg/ml) or rhIL‐8 (500 μg/ml) after incubation for 24 h in vitro with leg ulcer tissue extracts. Converse, IL‐1α, IL‐1β and IL‐8 levels increased several fold after 24 h compared with the 1‐h collection period with foam oblates.^5^ Obviously, the question of cytokine degradation lends itself to further research. To isolate the effects of proteolytic degradation, monitoring endogenous cytokine levels in WF incubated in vitro would be one procedure. Nonetheless, it would be valuable to compare NovaSwab to direct collection methods that require a short contact time with the wound bed for WF sampling, for example, paper or glass filters.^48^, ^49^

With the NovaSwab method it is possible to analyse small WF volumes and possibly dry wounds with ∼200 μl of diluted WF left for analysis. We compared the NovaSwab method to a similar technique that utilises undiluted WF to reduce analyte detection issues.^21^ Small but statistically significant differences between the Nova Swab and Schmohl methods were found in vitro for GM‐CSF, IL‐1α, IL‐6 and TNF‐α.

The NovaSwab method has potential for further optimization. The procured WFs were centrifuged to isolate extracellular WF from cells and cell debris (step 5, Figure 1), which might have damaged the cells and caused intracellular leakage of cytokines. This possibility could be studied by the release of lactate dehydrogenase. Apart from studying the effect of centrifugal force, optimization of the centrifugation time is needed. Thus, centrifugation conditions could be scrutinised by performing in vitro recovery experiments with naïve WF accumulated in foam oblates not subjected to centrifugation and freezing.

In conclusion, the six‐step NovaSwab method is noninvasive, easy to use and fast (≤15 min) with satisfactory recovery of the investigated cytokines in WF, and it is a promising research tool to monitor cytokine fluctuations during wound healing. Future larger studies should assess the interobserver variation and reproducibility of the new method.

## FUNDING INFORMATION

Kongelig Hofbuntmager Aage Bangs Fond provided funding for the multiplex assay kits.

## CONFLICT OF INTEREST

No company or organisation played any role in the initiation or design of the study, collection, analyses, or interpretation of the data, writing of the manuscript, or decision to publish the results. Reponex Pharmaceuticals sponsors Ewa Anna Burian's PhD studies through payments to the department. Ewa Anna Burian is investigating the effect of topical GM‐CSF in venous leg ulcers for Reponex Pharmaceuticals. Tonny Karlsmark is a medical advisor for Reponex Pharmaceuticals. Christian Enevold and Magnus S. Ågren declare no conflicts of interest.

## Supporting information


**Figure S1.** Interaction of cytokines in WF by the NovaSwab and Schmohl methods assessed in vitro. (A) Design of the experiment. WF was collected by foam oblates from the 12 patients and centrifuged. Cell‐free WF supernatants (50 μl) were added to the tip of the swab (∑‐Transwab [*n* = 11] and eSwab [*n* = 1]) and processed according to the NovaSwab and Schmohl methods or not (controls). (B) Cytokine concentrations were compared between the two methods and to WF alone using a two‐tailed paired *t*‐test. *p < .05, **p < .01.
**Figure S2**. Effect of saline irrigation on cytokine levels assessed by the NovaSwab method (pre and post saline). Open circles represent WF samples obtained by eSwab and closed circles by ∑‐Transwab. *p < .05, **p < .01 (two‐tailed paired *t*‐test).
**Table S1**. Luminex® Discovery Assay, used for determination of cytokine levels in the study. (Human Premixed Multianalyte Kit, R&D Systems, LXSAHM‐08).
**Table S2**. MSD, S‐plex Platform used for determination of GM‐CSF levels in the study. Kit 151F3S.

## Data Availability

The data that supports the findings of this study are available in the supplementary material of this article.
