# Supplementary material for: A simplified method for monitoring cytokines in wound fluid
Source: Wound Repair Regen. 2022 Oct 26;31(1):47–55. doi: 10.1111/wrr.13053 (PMC10092818; doi:10.1111/wrr.13053)
Supplement: Supplementary file 1 — Figure S1. Interaction of cytokines in WF by the NovaSwab and Schmohl methods assessed in vitro. (A) Design of the experiment. WF was collected by foam oblates from the 12 patients and centrifuged. Cell‐free WF supernatants (50 μl) were added to the tip of the swab (∑‐Transwab [n = 11] and eSwab [n = 1]) and processed according to the NovaSwab and Schmohl methods or not (controls). (B) Cytokine concentrations were compared between the two methods and to WF alone using a two‐tailed paired t‐test. *p < .05, **p < .01. Figure S2. Effect of saline irrigation on cytokine levels assessed by the NovaSwab method (pre and post saline). Open circles represent WF samples obtained by eSwab and closed circles by ∑‐Transwab. *p < .05, **p < .01 (two‐tailed paired t‐test). Table S1. Luminex® Discovery Assay, used for determination of cytokine levels in the study. (Human Premixed Multianalyte Kit, R&D Systems, LXSAHM‐08). Table S2. MSD, S‐plex Platform used for determination of GM‐CSF levels in the study. Kit 151F3S. [file WRR-31-47-s001.docx]

**Supplementary tables and figures**

**Table S1**. Luminex^®^ Discovery Assay, used for determination of cytokine levels in the study.

(Human Premixed Multianalyte Kit, R&D Systems, LXSAHM-08)

| Analyte | Standard curve (pg/ml) | Sensitivity (pg/ml) |
| --- | --- | --- |
| GM-CSF | 12.3 - 3000 | 4.1 |
| IL-1α | 4.94 - 1200 | 0.9 |
| IL-1β | 17.7 - 4300 | 0.8 |
| IL-6 | 4.53 - 1100 | 1.7 |
| IL-8/CXCL8 | 4.12 - 1000 | 1.8 |
| PDGF-AA | 4.94 - 1200 | 0.747 |
| TNF-α | 8.23 - 2000 | 1.2 |
| VEGF | 8.23 - 2000 | 2.1 |

**Table S2.** MSD, S-plex Platform used for determination of GM-CSF levels in the study. Kit 151F3S.

| Analyte | Dynamic range (fg/ml) | Sensitivity (fg/ml) |
| --- | --- | --- |
| GM-CSF | 1.9 - 26000 | 1.9 |


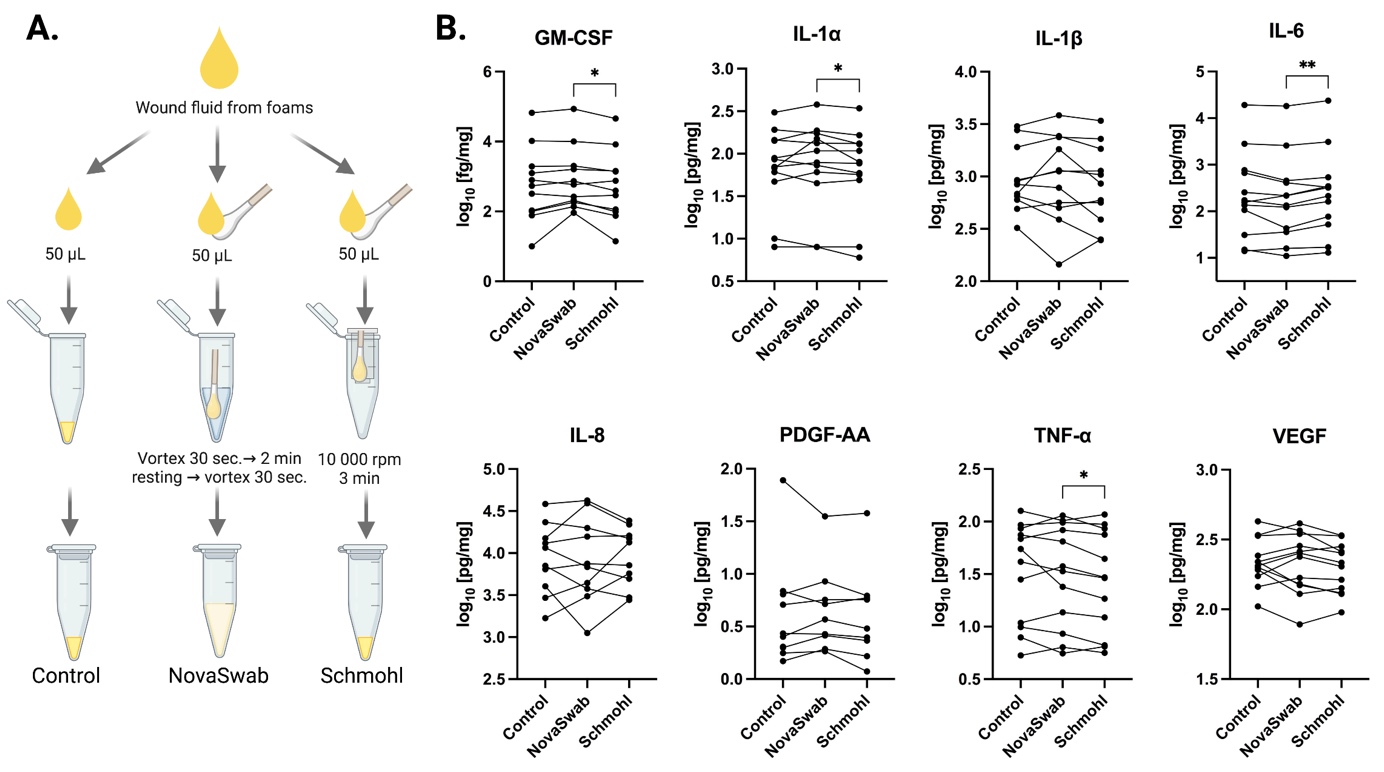


**Figure S1.** Interaction of cytokines in WF by the NovaSwab and Schmohl methods assessed *in vitro*. (A) Design of the experiment. WF was collected by foam oblates from the 12 patients and centrifuged. Cell-free WF supernatants (50 µl) were added to the tip of the swab (Σ−Transwab (*n* = 11) and eSwab (*n* = 1)) and processed according to the NovaSwab and Schmohl methods or not (controls). (B) Cytokine concentrations were compared between the two methods and to WF alone using a two-tailed paired t-test. ******p* < 0.05, ***p* < 0.01


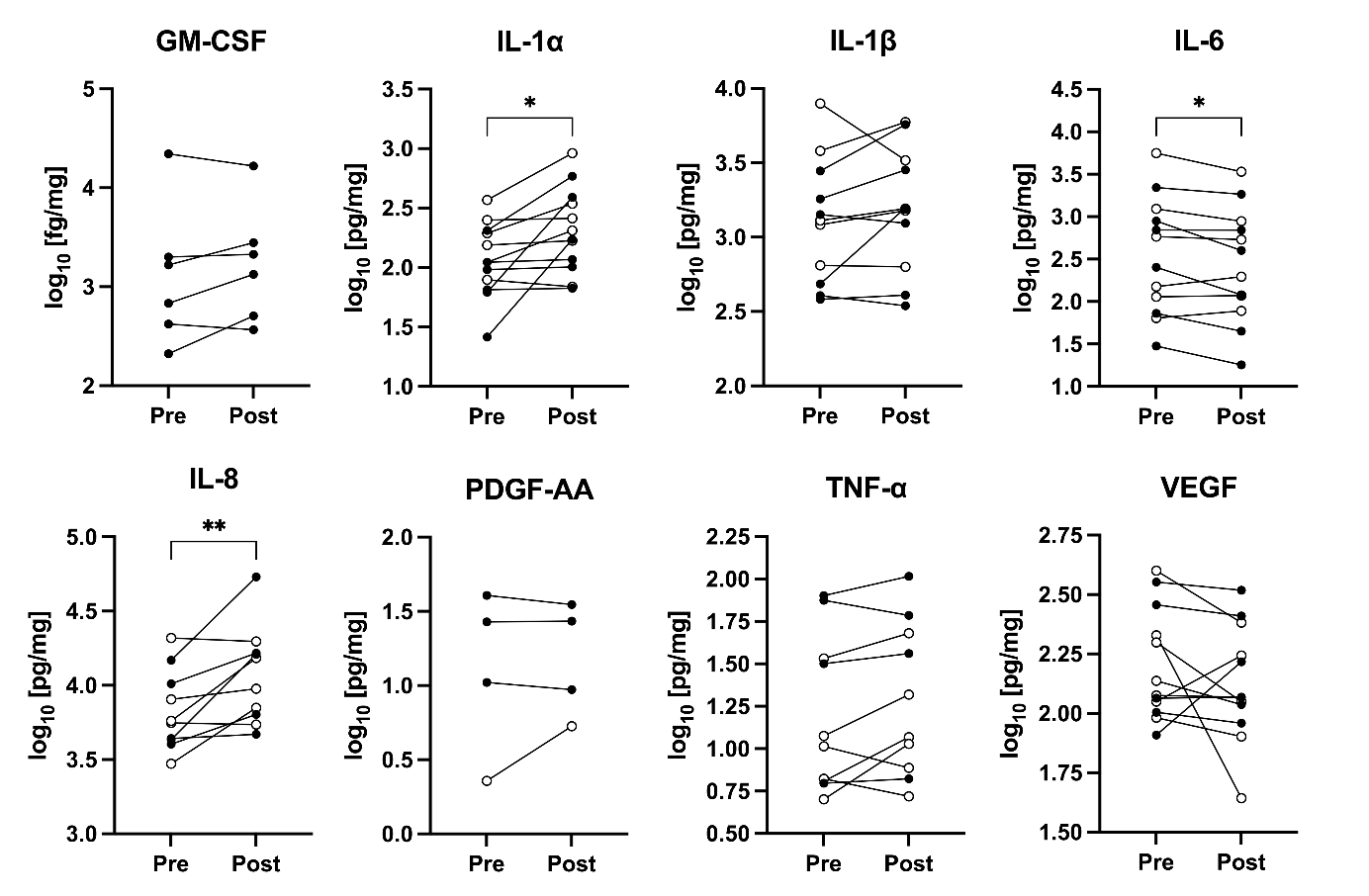


**Figure S2.** Effect of saline irrigation on cytokine levels assessed by the NovaSwab method (pre and post saline). Open circles represent WF samples obtained by eSwab and closed circles by Σ−Transwab. ******p* < 0.05, ***p* < 0.01 (two-tailed paired t-test).
